# Bronchopericardial Fistula: A Rare Complication of Necrotizing Pneumonia

**DOI:** 10.7759/cureus.64339

**Published:** 2024-07-11

**Authors:** Sameer N Bele, Manu M, Rakhi A Gosavi, Sanjay N Gaikwad

**Affiliations:** 1 Department of Pulmonary Medicine, Byramjee Jeejeebhoy (BJ) Government Medical College, Pune, IND

**Keywords:** cavity, tension pneumopericardium, broncho-pericardial fistula, klebsiella pneumoniae (kp), necrotising pneumonia

## Abstract

Pneumopericardium due to bronchopericardial fistula formation is a rare complication secondary to necrotizing pneumonia. Several such cases are reported due to different suppurative bacterial infections. Persistent fistulous communication has been reported to lead to tension pneumopericardium and hemodynamic instability, requiring urgent intervention such as pericardial drainage. A 41-year-old male patient, known to have chronic kidney disease and diabetes mellitus, presented with acute respiratory symptoms. Upon admission, the patient was febrile and required oxygen support via nasal prongs. A chest X-ray showed fibrocavitatory changes on the right side, with patchy air shadowing around the cardiac silhouette and a continuous diaphragm sign. A contrast-enhanced computed tomography (CECT) thorax revealed extensive areas of consolidation with necrotic areas within, forming a thin-walled cavity involving the right middle lobe. Also, suspicious communication of this cavity with the pericardial cavity along the right atrium was seen, with minimal pericardial collection and air foci within. The pleural fluid culture showed growth of *Klebsiella pneumoniae*. According to the antibiotic sensitivity report, the patient was started on IV meropenem and gentamicin for 21 days while monitoring kidney functions. The patient clinically improved on antibiotics, and follow-up radiological investigations showed resolution of pneumopericardium. In this patient, pneumopericardium was mild, and there was no evidence of tension pneumopericardium. Thus, conservative management with antibiotics was provided, with successful resolution. Unlike this case, if evidence of tension pneumopericardium had been present, emergency interventions for decompression would have been required, and these cases would have had a poor prognosis. This case demonstrates the importance of high suspicion and early diagnosis of pneumopericardium in patients with necrotizing pneumonia. Prompt treatment in these patients can prevent further life-threatening sequelae.

## Introduction

Pneumopericardium due to bronchopericardial fistula formation is a rare complication secondary to necrotizing pneumonia. A few such cases have been reported due to different suppurative bacterial infections in both immunosuppressed and immunocompetent patients. Persistent fistulous communication has been reported to lead to tension pneumopericardium, requiring urgent interventions such as pericardial drainage [1,2]. In contrast, mild pneumopericardium with no evidence of tension pneumopericardium can be successfully resolved with conservative management [2].

## Case presentation

A 41-year-old male patient presented to the ED with a history of dry cough, fever, and chest pain on the right side for onre month. He was a known case of diabetes mellitus and stable chronic kidney disease. Initially, the cough was associated with some expectoration, but it had been mostly dry for the past three weeks. The chest pain was mostly retrosternal in nature, and the fever was episodic, associated with chills and rigors. Upon admission, the patient’s pulse was 120 beats/min, blood pressure was 104/70 mmHg, temperature was 37.5° Celsius (99.5° F), and oxygen saturation was 94% on room air. The respiratory system examination showed reduced air entry on the right side with coarse crackles on auscultation.

**Figure 1:**
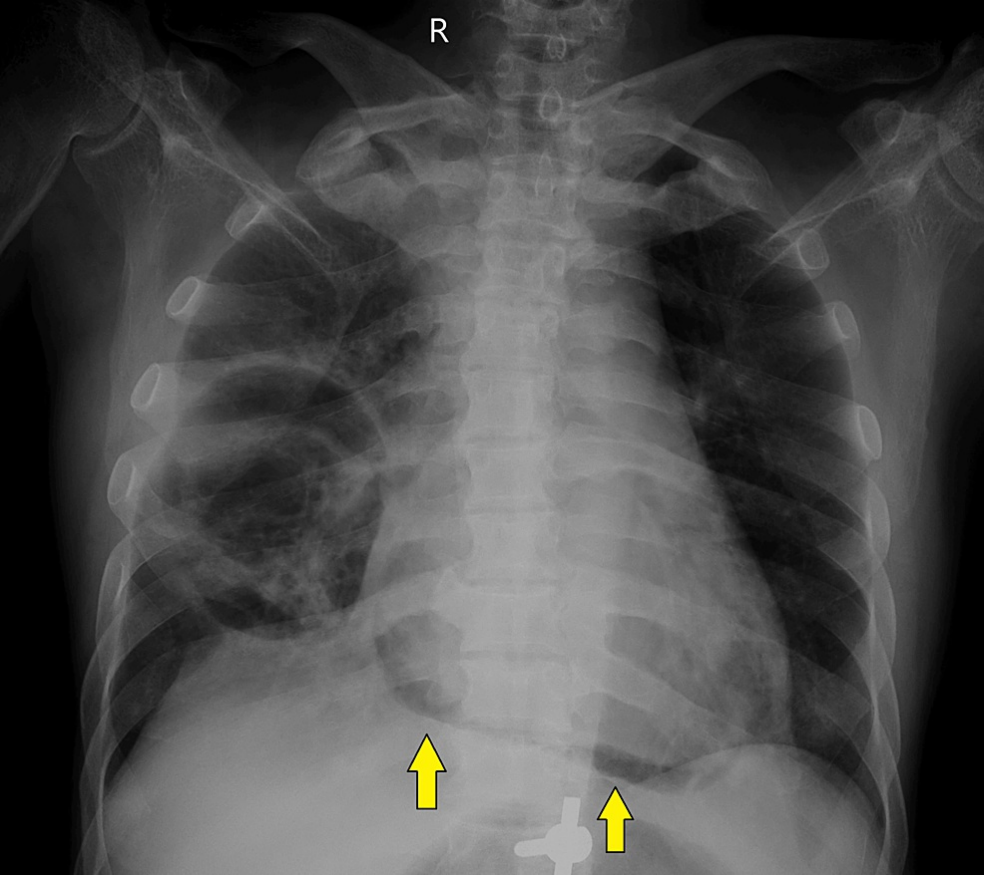
Chest X-ray of the patient on admission showing right middle zone cavitation and continuous diaphragm sign, pericardial air shadowing indicated by yellow arrows.

**Figure 2:**
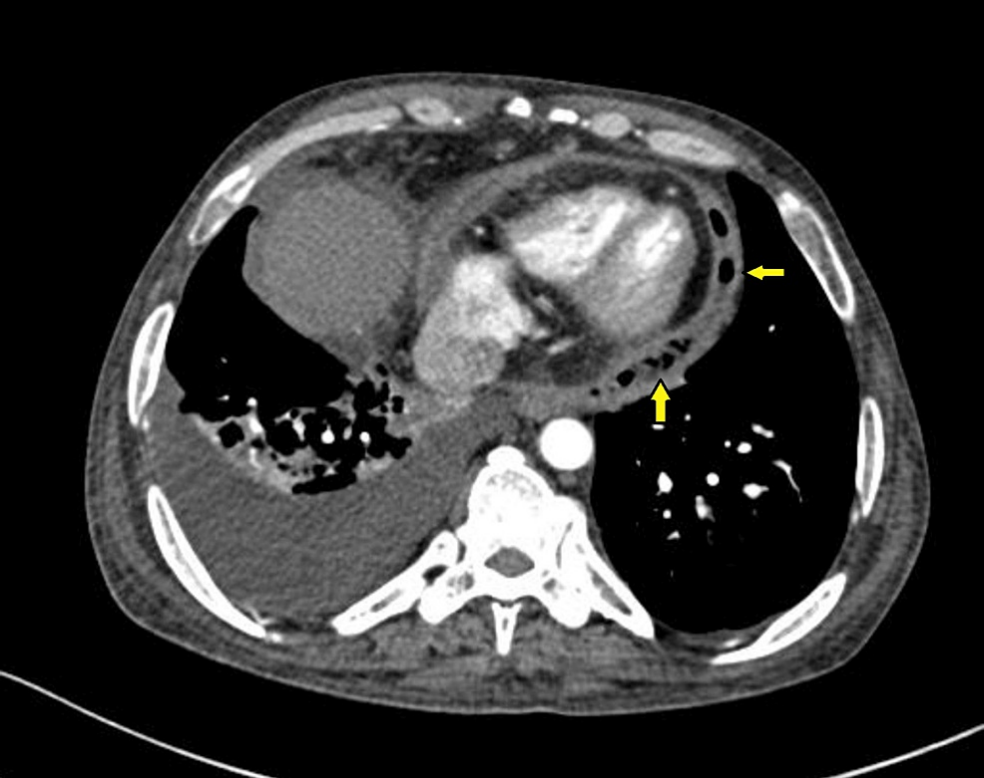
CECT thorax axial view section showing pericardial air foci suggestive of pneumopericardium, indicated by left arrows. Note the thickened pericardium. CECT: Contrast-enhanced computed tomography.

**Figure 3:**
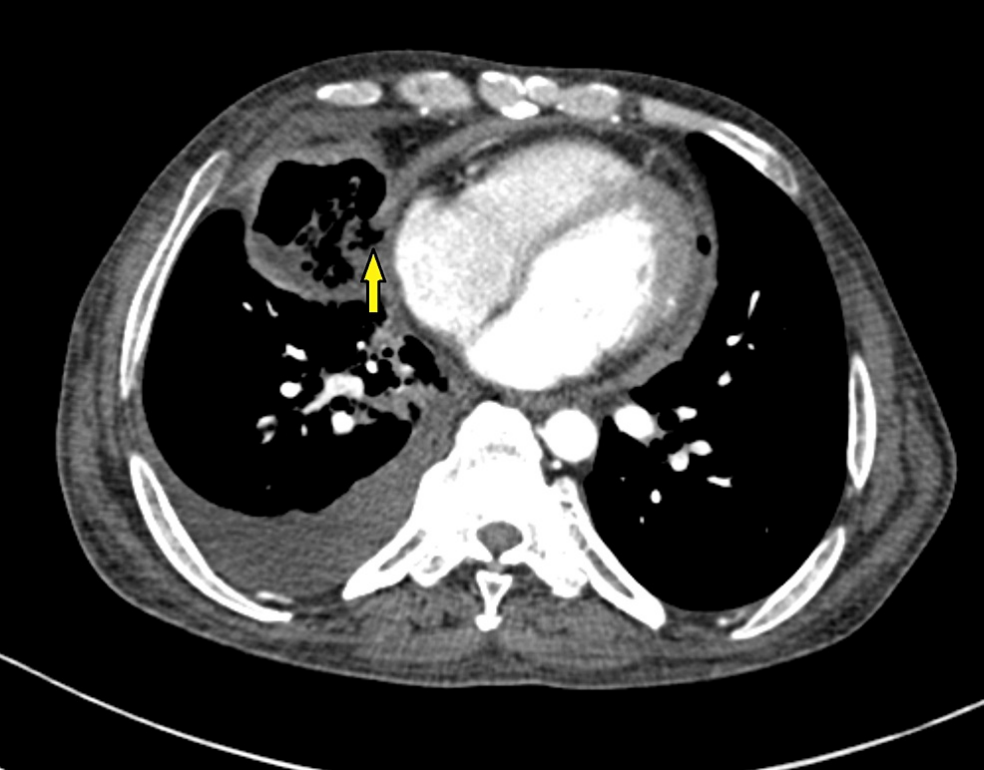
CECT thorax axial view section showing suspected fistulous communication of the right middle lobe cavity with the pericardial cavity along the right atrium, indicated by the left arrow. CECT: Contrast-enhanced computed tomography.

The chest X-ray, as shown in Figure 1, revealed fibro-cavitary changes on the right side with patchy air shadow around the cardiac silhouette and a ‘continuous diaphragm’ sign. His serum creatinine was 3.1 mg/dL, and his total leukocyte count was 14,500 cells/mm³ with a neutrophilic predominance (78%). A contrast-enhanced computed tomography (CECT) of the thorax showed extensive areas of consolidation with necrotic areas within, forming a thin-walled cavity involving the right middle lobe (3.9 x 5.7 x 5.9 cm) showing suspicious communication with the pericardial cavity along the right atrium with minimal pericardial collection and air foci within, as seen in Figures 2-3. The pericardium appeared to be thickened. The cavity also communicated with the right middle lobe bronchus. Additionally, he had multiple patchy ground-glass opacities forming consolidation involving the right upper lobe and bilateral lower lobes. Other findings included moderate right and minimal left-sided pleural effusion along with mediastinal lymphadenopathy. The 2D-Echo showed minimal pericardial effusion. USG-guided thoracentesis was performed on the right side, and pleural fluid culture showed growth of *Klebsiella pneumoniae*, sensitive to meropenem, tetracycline, and gentamicin, according to the antibiotic sensitivity report.

As there were no signs of tension pneumopericardium, interventional drainage for pneumopericardium was not required. After a discussion with the cardiology team, a decision for conservative management with intravenous antibiotics for *Klebsiella pneumonia* was finalized, with close monitoring of the patient’s vitals for any signs of tension pneumopericardium. As per the antibiotic sensitivity report, dual antibiotic coverage with meropenem and gentamicin was started for 21 days, while monitoring renal function. The patient clinically improved with the resolution of cough and fever. Radiological improvement was also seen on chest X-ray and high-resolution CT (HRCT) thorax, with a reduction in the size of the cavity and resolution of pericardial air shadowing. The follow-up 2D echo showed normal cardiac function with normal pericardial movement, and the patient was discharged on oral antibiotics.

## Discussion

In this patient, pneumopericardium was mild and there was no evidence of tension pneumopericardium. Thus, conservative management with antibiotics was provided, resulting in a successful resolution.

Although penetrating, blunt, or iatrogenic traumas and carcinomas are common causes of pneumopericardium [2,3], this patient had no history of trauma, interventions, or malignancy to suspect the same. Initial suspicion of pneumopericardium was raised by the chest X-ray and confirmed by CT thorax. A few cases have been reported in journals of patients with infective etiologies such as necrotizing bacterial pneumonia, histoplasmosis, and invasive pulmonary aspergillosis [2,3]. Common bacterial infections causing necrotizing pneumonia like *Staphylococcus aureus*, tuberculosis, and *Acinetobacter baumannii* infections have been shown to cause bronchopericardial fistulas [1,2,4,5]. Although well-known for causing necrosis, only a few cases of pneumopericardium are reported with *Klebsiella pneumoniae* pneumonia [6,7].

This complication, although rare, is more commonly seen in immunocompromised patients than in immunocompetent patients with pneumonia, due to aggressive necrosis [2]. This patient was also immunocompromised, having pre-existing diabetes mellitus and chronic kidney disease. These patients need close monitoring for tension pneumopericardium, manifested as tachypnea, increased jugular venous pressure with hypotension, and hemodynamic collapse due to cardiogenic shock [8,9]. Emergency pericardial drainage by pericardiocentesis and continuous drainage by pericardial pigtail catheter insertion are warranted if tension pathology is suspected. Although a clinical diagnosis, tension pneumopericardium can be demonstrated through dye leak in the pericardium and inspiratory column movement in the pericardial pigtail. These patients have a poor prognosis with high mortality rates. Hence, high suspicion of bronchopericardial fistula during clinical and radiological examinations, especially in immunocompromised patients with necrotizing pneumonia, should be routinely practiced.

## Conclusions

Our case demonstrates the importance of high suspicion and early diagnosis of pneumopericardium in patients with necrotizing pneumonia. Prompt treatment in these patients can prevent further life-threatening sequelae, as it is associated with high mortality.
